# Strigolactone GR24 modulates citrus root architecture and rhizosphere microbiome under nitrogen and phosphorus deficiency

**DOI:** 10.1186/s12870-025-07515-5

**Published:** 2025-11-14

**Authors:** Sabry Soliman, Alaaeldin Rezk, Fernando Igne Rocha, Jean Carlos Rodriguez-Ramos, Bharani Manoharan, Yi Wang, Zhenhai Han, Lauren Hale, Ashraf El-kereamy

**Affiliations:** 1 Department of Botany and Plant Sciences, University of California Riverside, Riverside, CA 92521 USA; 2 Department of Horticulture, Faculty of Agriculture, Ain Shams University, Cairo, Egypt; 3 USDA, Agricultural Research Service, San Joaquin Valley Agricultural Sciences Center, 9611 South Riverbend Avenue, Parlier, CA USA; 4 Department of Fruit Science, College of Horticulture, China Agriculture University, Beijing, 100083 China

**Keywords:** Strigolactone, GR24, Root Architecture, Rhizosphere Microbiome, Nutrient deficiency, Citrus

## Abstract

Nutrient deficiencies, especially nitrogen (N) and phosphorus (P) deficiencies, are among the most critical challenges for sustainable crop production. Excessive use of chemical fertilizers contributes to environmental degradation and climate change, highlighting the need for alternative strategies to improve nutrient uptake. Strigolactones (SLs), a class of plant hormones, have emerged as key regulators of root development and plant–microbe interactions under nutrient-limited conditions. In this study, we investigated the effects of the SL analog GR24 on citrus rootstock C-32 under full nutrition, nitrogen deficiency, and phosphorus deficiency conditions. Plants were treated with five GR24 concentrations (0, 1, 2.5, 5, and 10 µM) and evaluated for changes in root architecture, biomass, soil nutrient content, and rhizosphere microbiomes. The results showed that SL application had a concentration-dependent effect on root morphology. The 2.5 µM SL treatment enhanced fine root initiation, proliferation, and lateral branching under nutrient deficiency, whereas higher concentrations (5 and 10 µM) generally had inhibitory effects. Root surface area and volume were modulated differently across diameter classes, depending on nutrient status and SL dose. Soil nutrient analyses indicated minor alleviating effects of SL on N and P starvation; however, SL significantly affected Cu and Mn. Microbiome analysis revealed that SL reduced bacterial ASV richness but increased dispersion in community structure, particularly under N and P deficiency. Functional annotation indicated changes in denitrification and methanotrophy pathways. Overall, SLs modulated both the root architecture and rhizosphere microbiome composition of citrus plants under nutrient stress. These findings suggest a dual role for SLs in enhancing root plasticity and microbial recruitment, with potential applications in sustainable crop nutrition and soil health.

**Supplementary Information:**

The online version contains supplementary material available at 10.1186/s12870-025-07515-5.

## Background

Nutrient deficiencies, particularly nitrogen (N) and phosphorus (P) deficiencies, are among the most limiting factors for global crop productivity and pose significant challenges to sustainable agriculture [1–6]. To compensate for these deficiencies, large quantities of chemical fertilizers are often applied, which not only increase production costs but also contribute to environmental degradation and other environmental crises, such as greenhouse gas emissions, which increase global warming and contribute to climate change [1, 3–8]. Therefore, developing strategies that increase nutrient uptake efficiency and reduce dependence on chemical inputs is critical [6, 9–22].

Strigolactones (SLs) are a relatively recently discovered class of carotenoid-derived plant hormones that have been shown to play multifunctional roles in root development and stress responses [14, 16, 18, 19, 21, 23–36]. In addition to regulating shoot branching, SLs are increasingly recognized for their involvement in root system architecture modulation and plant interactions with beneficial soil microbes, particularly under nutrient-deficient conditions [15, 17, 25, 37]. By influencing root elongation, lateral root formation, and fine root proliferation [38, 39], SLs may increase a plant’s capacity to explore soil volumes more effectively for nutrient acquisition. Furthermore, SLs have been implicated in shaping the composition and activity of rhizosphere microbiomes [35, 40, 41], potentially influencing nutrient cycling and availability [33, 34, 42, 43].

In this study, we investigated the effects of the synthetic SL analog GR24 on the growth and rhizosphere ecology of the citrus rootstock C-32 subjected to three nutrient regimens: full nutrition, nitrogen deficiency, and phosphorus deficiency. GR24 was applied at five concentrations (0, 1, 2.5, 5, and 10 µM) to examine dose-dependent responses. We evaluated changes in root system architecture, including root length, surface area, volume, and diameter distribution, as well as total biomass accumulation. In addition, we analyzed the soil nutrient content, with a focus on N and P availability, and assessed shifts in rhizosphere bacterial communities via amplicon sequencing.

Our results demonstrated that GR24 modulates root development in a concentration- and nutrient-dependent manner, with 2.5 µM showing the greatest positive effects on fine root proliferation and lateral branching under nutrient-deficient conditions. Higher concentrations generally exhibited inhibitory effects. The GR24 treatment also had subtle effects on soil N and P availability but significantly influenced micronutrient dynamics, particularly copper (Cu) and manganese (Mn) contents. Moreover, GR24 application altered rhizosphere microbial diversity and structure, reducing ASV richness but increasing community dispersion, especially under N and P deficiency. The functional annotation of the microbial communities suggested the modulation of key pathways, such as denitrification and methanotrophy.

These findings highlight the potential of SLs as biostimulants capable of enhancing root plasticity and beneficial microbial interactions under nutrient stress. The dual impact of SLs on plant morphology and rhizosphere function underscores their relevance as tools for improving nutrient use efficiency and promoting more sustainable crop production systems.

## Materials and methods

### Experimental conditions

The experiment was carried out in a greenhouse located at the University of California, Lindcove Research and Extension Center, Exeter, California. The experiment was conducted using C-32 citrange trifoliate hybrid rootstock seedlings (*X Citroncirus* spp.). This rootstock was developed in 1953 by the University of California, Riverside breeding program as a hybrid between ‘Ruby’ sweet orange and ‘Webber-Fawcett’ trifoliate. C-32 is publicly available through the Citrus Clonal Protection Program (CCPP) (https://ccpp.ucr.edu/variety/287.html) under Variety Index Number (VI# 287). Seeds were harvested from 20-year-old trees located in the foundation block of the CCPP at the University of California Lindcove Research and Extension Center, Exeter, California. Additional information regarding the origin of C-32 is provided by the Givaudan Citrus Variety Collection at the University of California, Riverside (https://citrusvariety.ucr.edu/crc3911). The environmental conditions were recorded during the experimental period by a humidity/temperature portable data logger (Model RHT20, EXTECH INSTRUMENTS, 15, Flir LLC). The average temperature was 20–35 °C, and the relative humidity was 40–60% under a normal photoperiod from May to July, about 12–15 h.

### Plant materials and planting

The seedlings used in the experiment were 4-month-old C-32 rootstock. These were grown at Ray Leach “super cell” UV (cones). The top diameter was 4 cm, and the depth was 20 cm. Then it was transferred to other pots, which were 12.5*12.5 in width * length, and the depth was 13.5 cm.

### Growth media and conditions

The growth media contained 100% peat moss. The C-32 seedlings were grown in the plastic pots containing 100% peat moss as a growth medium. The peatmoss was analyzed to ensure a low content of N or P by Fruit Growers Laboratory, Inc., an analytical environmental service company at Visalia, California.

### Experimental design

The experiment was factorial and consisted of two factors. The first factor was nutrient deficiency or starvation at three levels: full fertilization, N deficiency, and P deficiency. The second factor was the application of the phytohormone strigolactone (GR24), which has five different concentrations. The experiment followed a split-plot design (SPD), with nutrition assigned to the main plots and GR24 applications to the subplots. Main plot effects were tested against the main plot error term, whereas subplot effects and their interaction with the main plot factor were tested against the subplot error term. The plants were divided into three experimental groups (first factor). The first group of plants was fully fertilized, the second group was fertilized with N, and the final group of plants was subjected to P starvation. then this groups treated by spraying GR24 concentrations (second factor).

Each main plot treatment was replicated 3 times, with 4 subplot replicates per main plot. Variation was partitioned into main plot error, subplot error, and residual error, consistent with split-plot design requirements. This comprises 180 plants in total, with 60 plants in each experimental group. The concentrations of GR24 used were 0, 1, 2.5, 5, and 10 µM, with 15 plants used for each concentration. The N and P starvation groups of plants were sprayed with GR24 every two days, and the application was carried out 3 times via a hand sprayer. The plants were sprayed every two days by a hand sprayer until the solution covered the entire leaves and the solution trickled down the leaves [44].

### Nutrient treatments and irrigation

The transplants were irrigated every day and sprayed with filtered water to ensure that there was no source of nutrients. All the transplants were regularly irrigated every two days in the summer season. After the treatments were started, distilled water was used for irrigation, and nutrient application was performed with Hoagland solution. The nutritional profile used during the experiment was determined according to the formula of Hoagland [45, 46]. Molar stock solutions for each nutrient element were prepared. The N: P:K ratio was 3:0.5:3.5, and the trace concentrations of micronutrients per g/L were added in the same manner as the Hoagland formula [45, 46] with the molar concentrations mentioned above. The nitrogen and phosphorus contents were also depleted, and the samples were prepared in the same way as the Hoagland solution. Plants received Hoagland nutrient solution (Full) containing 6.0 mM KNO₃, 4.0 mM Ca(NO₃)₂, 1.0 mM KH₂PO₄, 2.0 mM MgSO₄, 0.10 mM Fe (as Fe-EDTA), and micronutrients (0.046 mM H₃BO₃, 0.009 mM MnCl₂, 0.00077 mM ZnSO₄, 0.00032 mM CuSO₄, 0.00019 mM Na₂MoO₄). The –N solution replaced KNO₃ and Ca(NO₃)₂ with equimolar KCl (6.0 mM) and CaCl₂ (4.0 mM). The –P solution omitted KH₂PO₄ and added 1.0 mM KCl to maintain K⁺. Solutions were adjusted to pH 5.8–6.0 before application. Different bucket tanks were used to separate the stock solutions and the starvation stock solutions. Distilled water was used to dissolve the pure salts to prepare the full nutrient stock solution. The nitrogen and phosphorus salts were replaced with other salts to provide other nutrient elements without changing the balance of the nutrient stock solutions, the absence of other nutrient elements or the EC of the solution, which was between 1.42 and 2.76 mS/cm. Each plant received 150 ml of each solution, and the treatment was repeated every 3 days.

### Sample Preparation

In this study, the soil samples were analyzed for nutrient content to ensure a low level of N or P in each treatment. Five samples of soil, which were peat moss media with full fertilization and N and P deficiencies of 0 and 2.5, were collected. The concentration of 2.5 was selected, as it provided the most favorable morphological and vegetative responses compared with other tested concentrations. The collected soil samples were stored in the freezer at −20 °C and then used as backup samples and to investigate the macro- and micronutrient changes after treatment, while a baseline measurement was used in the soil analysis before the experiment began. Some samples from each treatment were randomly selected for nutrient analysis. Fifty grams of each representative sample were weighed and dried in an oven for seven days at 150 °C. The dry weights of the samples were measured, and three samples were subsequently sent to Fruit Growers Laboratory, Inc., an analytical environmental service company in Visalia, California, for full nutritional content analysis.

### GR24 Preparation and application

Plants were treated with rac-GR24, which contains mixed stereoisomers and is commonly used to study SL-related responses. (rac)-GR24 was used as an SL analog from PhytoTech Labs, Inc., USA. Five milligrams of (rac)-GR24 powder was dissolved in 85 µl of 0.5% DMSO and mixed well by vortexing, followed by the addition of 170 µl of acetone. The prepared solution was transferred to a 50 ml centrifuge tube containing 7.5 ml of 99% ethanol. Finally, distilled water was added to a volume of 17 ml, which served as a stock solution at a concentration of 10 µM. The stock solution was stored in a −20 freezer until use. Various volumes of 0, 450, 1125, 2250, and 4500 µL were pipetted from the stock solution at the time of application to obtain the desired concentrations of 0, 1, 2.5, 5, and 10 µM, respectively, for different treatments. Five groups of plants were subjected to full fertilization, nitrogen deficiency, and phosphorus deficiency. The first group was sprayed at 0 µM, the second was sprayed at 1 µM, and the same, along with others, was sprayed at up to 10 µM. In this solution, 0.1% Tween 20 was added to decrease the water surface tension and increase the absorption. The GR24 preparation was carried out at room temperature according to these methods [44, 47].

### Root characteristics and architecture analysis

The root length, surface, distribution, and diameter (ranging from 0 to 5 mm) were analyzed via a root scanner (LA Scanner 2400, Regent Instruments Inc.). This scanner is calibrated for image analysis with REGENT INSTRUMENTS software (WinRHIZO, Arabidopsis 2021a, 64-bit).

### Microbiome profiling

#### Soil DNA extraction, 16 S rRNA and ITS gene amplicon sequencing, sequencing data processing, and microbiome statistical analysis

Three plants from each of the full, N-deficient, and P-deficient fertilizer treatments with and without foliar spray (0 or 2.5 µM GR24) (*n* = 24) were used to profile the root surface microbiomes. Rhizosphere soil DNA was extracted via a modified version of a published protocol (Edwards 2015) [48] in which the roots were washed in a solution of PBS + 0.05% Tween. After the rhizosphere soil was pelleted, the soil DNA was extracted via a DNeasy PowerSoil Pro DNA extraction kit (Qiagen) following the manufacturer’s instructions. For each sample, 12.5–17.5 ng of DNA was used to prepare triplicate reactions of a two-step PCR workflow (Illumina) [49], with primers 515f and 806r [49, 50] used in the first step. Amplicon libraries were quantified via a Quant-IT PicoGreen ds-DNA specific reagent (Invitrogen), pooled at equal molality, gel purified (Qiaquick Gel Extraction Kit), and validated for accurate amplicon length via a bioanalyzer. Pooled libraries were sequenced at the UC Davis Genome Center on an Illumina MiSeq instrument. The processing of 16 S rRNA and ITS genomic libraries was conducted via the sequence analysis tool within the IEG Data Management Pipeline (http://ieg3.rccc.ou.edu:8081/) following the DADA2 workflow. After generating abundance tables of amplicon sequence variants (ASVs) and taxonomic annotations via Silva 138.1 for 16 S rRNA and the UNITE classifier v9.0 for ITS, microbial ecology analyses were performed via a suite of packages in the R environment, including phyloseq (McMurdie et al., 2013) [51], ampvis2 (Andersen et al., 2018) [52], microeco (Liu et al., 2021) [53], and their dependencies. Functional prediction analysis was conducted via the FAPROTAX database (Louca et al., 2016) [54] to assess the impact of nutrient conditions and SL application on bacteriome functions. Finally, a correlation-based network analysis was carried out to evaluate the topological features of the bacterial and fungal communities across treatments combining nutrient conditions with and without SL application. Only significant associations (*p* < 0.01) and correlations above 0.70 were included. Aesthetic attributes, layout, and topologies were determined in Gephi v0.9.2. Node sizes represent betweenness centrality values, and colors indicate module affiliation.

### Statistical analysis

Data were analyzed using a split-plot ANOVA in CoStat software, version 6.303 (CoHort Software, Monterey, CA, USA). Model assumptions of normality and homogeneity of variances were verified using Bartlett’s Test, and diagnostic residual plots.Where necessary, data were log-transformed to meet assumptions. Statistical analyses were performed separately for root architecture traits (e.g., total length, surface area, volume, and root tips), root biomass (fresh and dry weight), and soil nutrient content under different nutrient and strigolactone (SL) treatments. A split-plot ANOVA design was used, with nutrient conditions (full, –N, and –P) as the main plot factor and SL concentrations (0, 1, 2.5, 5, and 10 µM) as the subplot factor. The treatment means were compared via Duncan’s multiple range test at *p* < 0.05.

## Results

### Effects of Strigolactone on total root architecture parameters, fresh weight, and dry weight

**Fig. 1:**
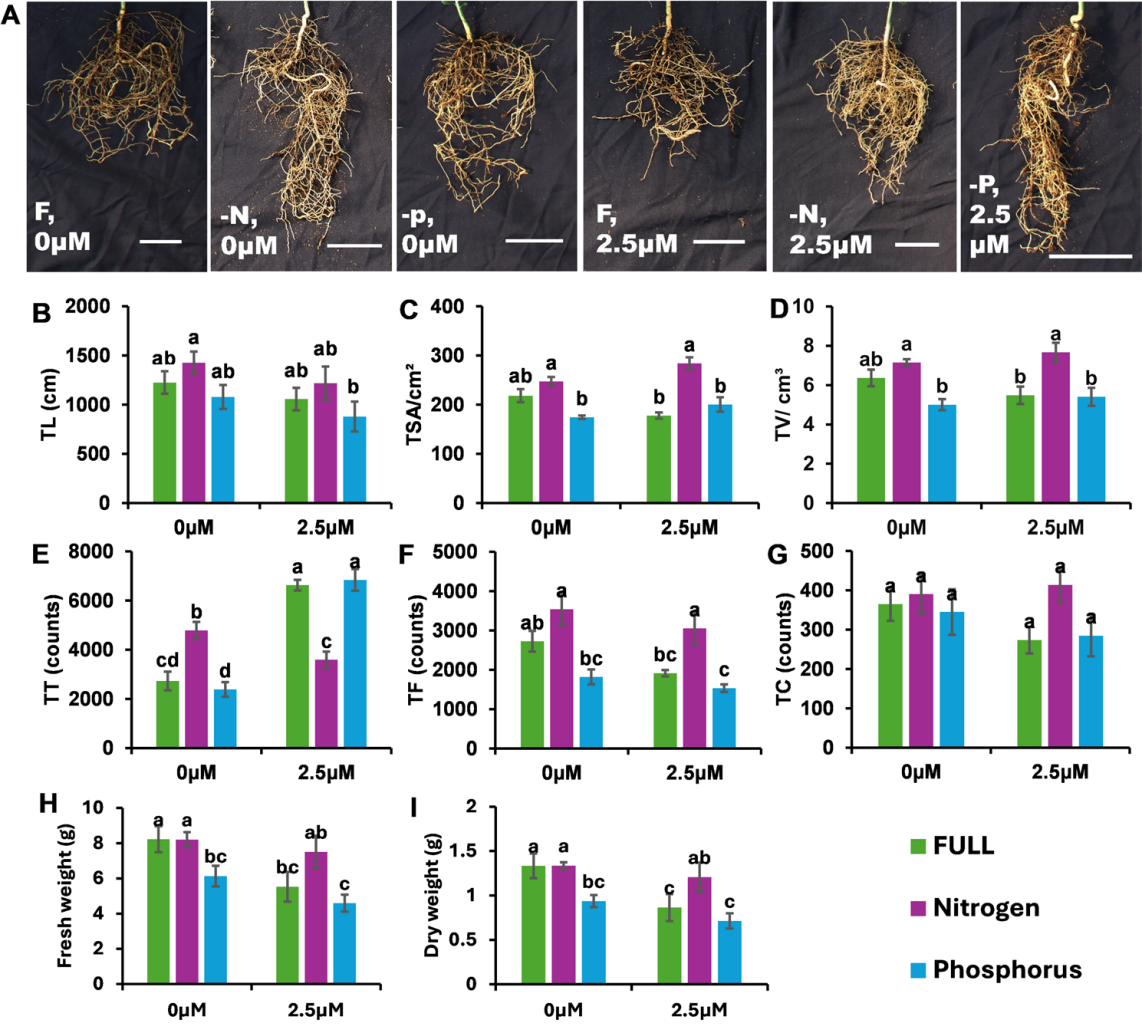
Effects of strigolactone (SL) application on total root architecture and biomass parameters in Citrus C-32 rootstock under full nutrition, nitrogen deficiency, and phosphorus deficiency conditions. **A** and **B** Total root length (TL), (**C**) total root surface area (TSA), (**D**) total root volume (TV), (**E**) number of root tips (Tips), (**F**) number of root forks, (**G**) number of root crossings, (**H**) root fresh weight (FW), and (**I**) root dry weight (DW). The SL concentrations tested were 0 and 2.5 μM. The bars represent the means ± standard errors (SE). Different lowercase letters indicate statistically significant differences among treatments (*p* < 0.05)

The application of 2.5 µM strigolactone (SL) under various nutritional conditions significantly influenced root architecture and nutrient accumulation. Under full nutrient conditions, the SL treatment significantly increased the number of total root tips (TTs). Under phosphorus starvation, SLs also promoted TT. Conversely, under nitrogen starvation, SL application resulted in a significant decrease in the total number of root tips. Figure 1.

Interestingly, SLs negatively influenced overall root biomass under full nutrient conditions. Compared with those of the untreated control plants, both the fresh weight and dry weight (FW, DW) of the roots of the SL-treated plants were significantly lower. These reductions were not reported under N or P starvation, suggesting that the biomass-related responses to SLs may be specific to the availability of complete nutrients. Figure 1.

**Fig. 2:**
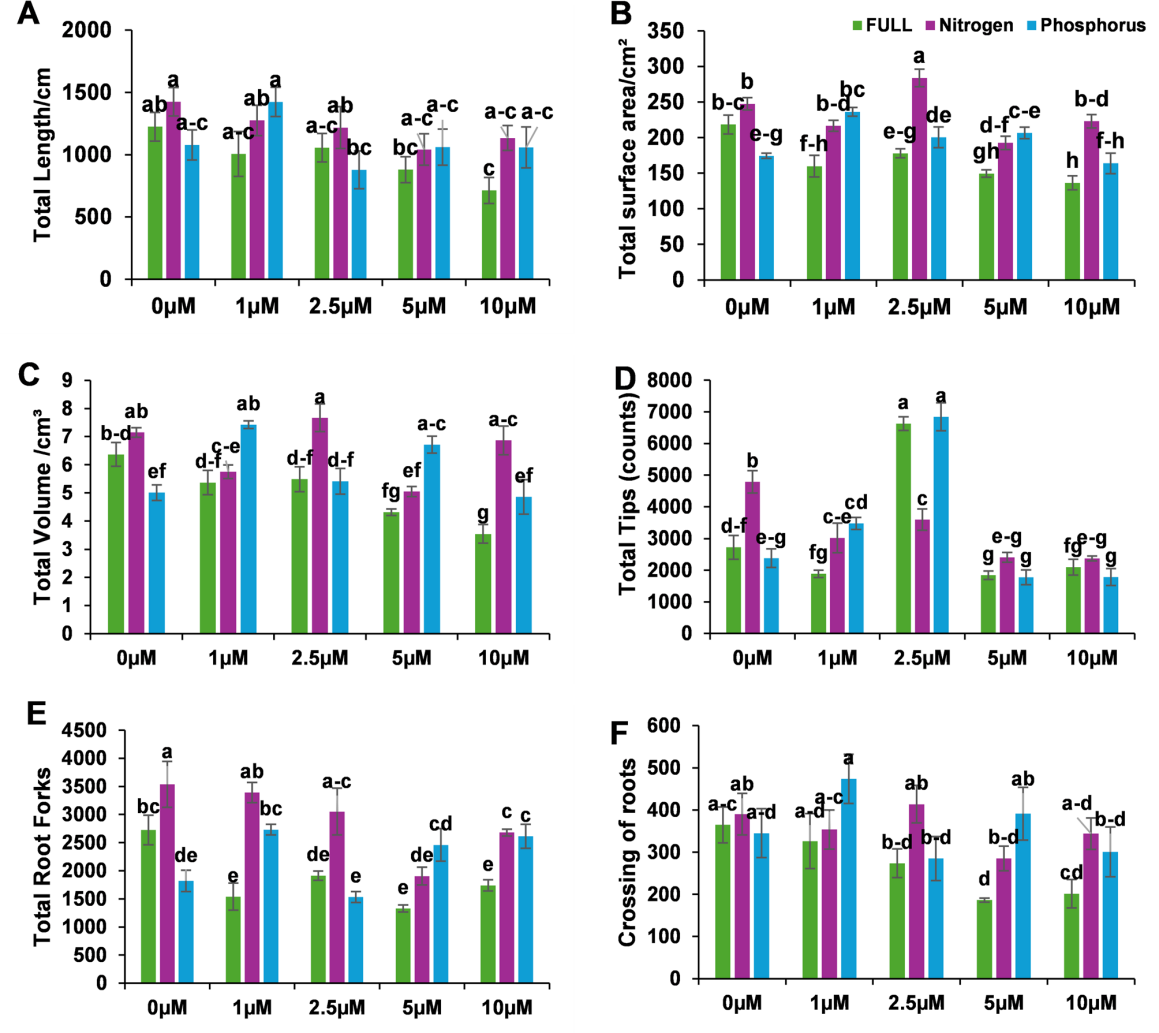
Summary of total root architectural parameters under SL application at 0, 1, 2.5, 5, and 10 µM across full nutrition, nitrogen deficiency, and phosphorus deficiency conditions. Parameters include: **A** total root length (TL), **B** surface area (TSA), **C** volume (TV), **D** number of root tips, **E** forks, and **F** crossings. Bars represent means ± SE. Different lowercase letters indicate statistically significant differences among treatments (*p* < 0.05)

At 2.5 µM SL, no significant differences were observed in other overall root architectural traits—including total length (TL), total surface area (TSA), total volume (TV), total forks (TF), and total cross (TC)—under either nitrogen or phosphorus deficiency. In contrast, higher SL concentrations (5 and 10 µM) exerted inhibitory effects on root development, as evidenced by reductions in TL, TSA, and TV **(**Fig. 2**)** For example, at 10 µM SL, root total length was significantly decreased under full nutritional conditions compared with other treatments, suggesting a negative or growth-suppressive effect at elevated SL levels. Conversely, a low concentration (1 µM SL) significantly enhanced TSA and TV, indicating a stimulatory role at this level. Notably, 2.5 µM SL produced the highest TSA values across all treatments, surpassing 0, 1, 5, and 10 µM SL, thereby suggesting that this concentration represents an optimal level for promoting root surface development in studied citrus **(**Fig. 2**)**.

#### The effects of strigolactone on the root surface area (SA) and root volume (V) per diameter ranged from 0.5 to over 5 mm

**Fig. 3:**
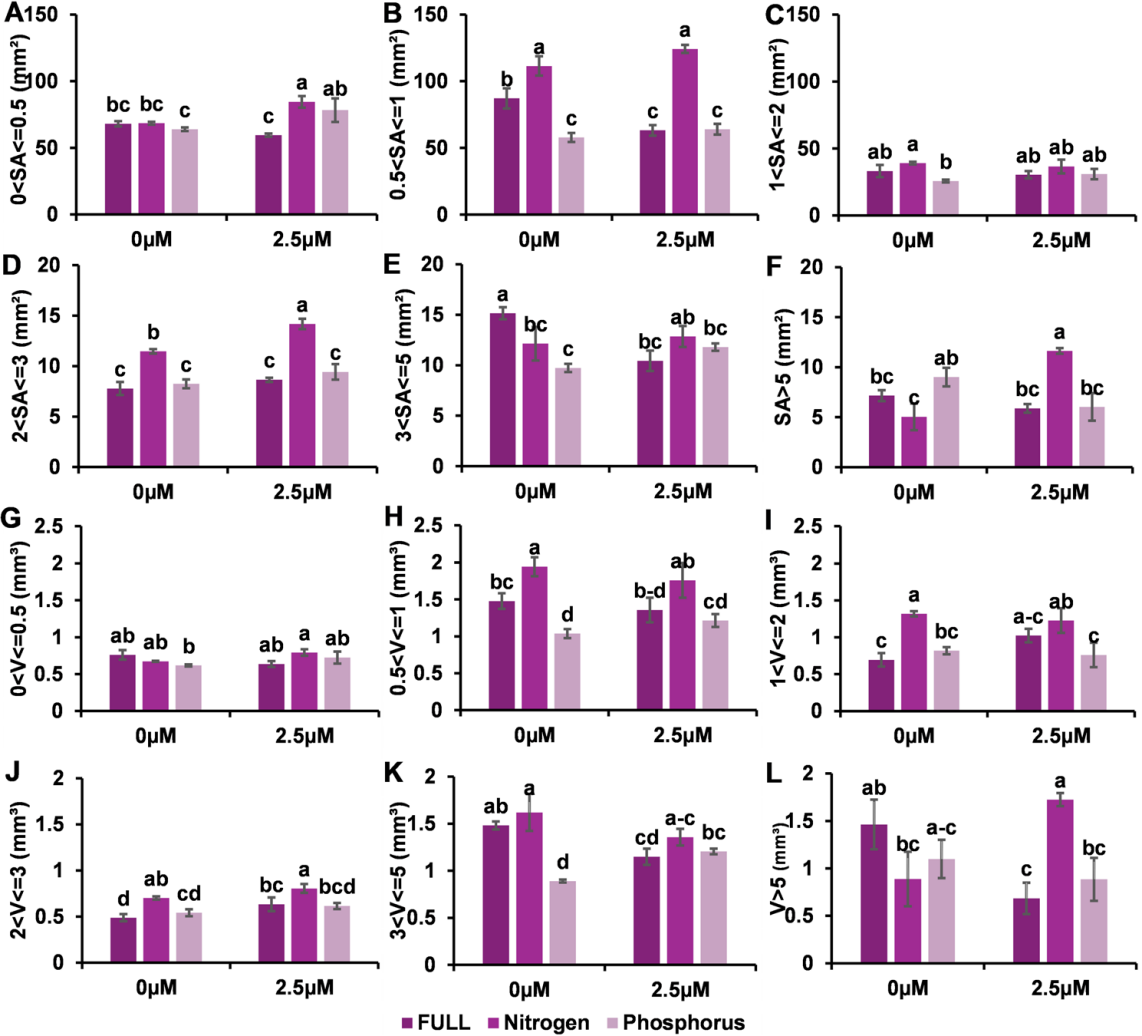
Effects of SLs on root surface area (SA) and volume (V) across root diameter classes under full nutrition, nitrogen, and phosphorus deficiency. The root surface area is shown in panels (A–F) for different diameter ranges: **A** 0–0.5 mm, **B** 0.5–1 mm, **C** 1–2 mm, **D** 2–3 mm, **E** 3–5 mm, and **F** > 5 mm. The root volume is shown in panels **G**–**L** for the same diameter ranges. The bars represent the means ± SEs. Different letters indicate significant differences between treatments (*p* < 0.05)

The response of the root surface area (SA) to SL treatment varied depending on the nutrient conditions. Under full nutritional conditions, SLs decreased the surface area of both the fine roots and the root hairs, as well as the coarse roots of the main and lateral roots. However, under nitrogen starvation, SL application significantly increased the surface area of very fine and root hairs, coarse roots, and very coarse main and lateral roots (diameters 0–0.5.5, 2–3, and over 5 mm^2^. Similarly, under phosphorus starvation, SL promoted an increase in the surface area of very fine roots and root hairs. (Fig. 3 and supplementary Fig. 1)

The root volume was also differentially affected by SL treatment. In fully nourished plants, SLs decreased the volume of coarse and very coarse roots of the main and lateral roots. In contrast, nitrogen-starved plants presented an increase in the volume of very coarse roots following SL treatment. Similarly, phosphorus-starved plants presented an increase in the volume of coarse roots of the main and lateral roots after SL application. (Fig. 3 and supplementary Fig. 2)

### The effects of strigolactone on root length (RL) and tips (T) per diameter ranged from 0.5 to over 5 mm

**Fig. 4:**
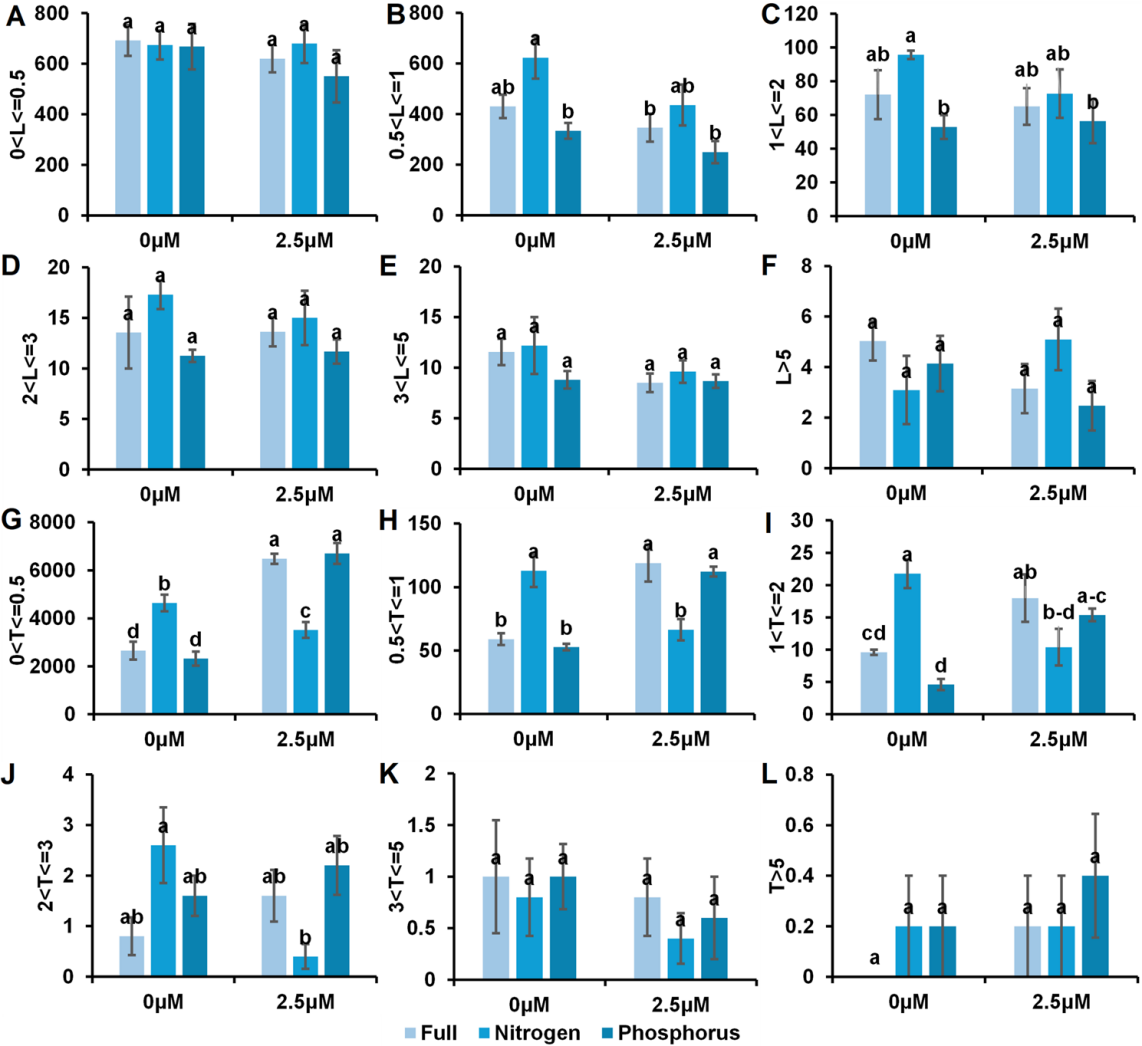
Effects of SL application on root length (RL) and the number of root tips (T) across diameter classes in Citrus C-32 rootstock under nutrient treatments. Panels (**A**–**F**) show root length for roots 0–0.5 mm to > 5 mm in diameter. Panels (**G**–**L**) show root tip counts in the same ranges. The full results are shown in Supplementary Figs. 2 and 5. The bars represent the means ± SEs. Different letters indicate statistically significant differences (*p* < 0.05)

SL application at 2.5 µM significantly affected root tip development under different nutritional regimens. Under full nutrient conditions, the SL treatment increased the number of very fine roots and root hairs, in addition to medium-sized lateral roots. In phosphorus-starved plants, SLs increased the number of root tips in very fine, fine root hairs and medium-sized lateral roots. Conversely, under nitrogen starvation, SL application resulted in a marked reduction in the number of root tips across all categories, including very fine, fine, and medium-sized lateral, coarse, and very coarse roots. In contrast, higher concentrations (5 and 10 µM SL) had no significant effect on tips at various root diameter ranges. **(**Fig. 4, and Supplementary Fig. 3)

Although the length of roots in different diameter classes (from 0 to greater than 5 mm) varied among the treatments, the differences were not statistically significant. In addition, the longest roots were observed in the N starvation treatment. Although 2.5 µM SL did not significantly differ, 1 µM SL alleviated the reduction in root length caused by N and P starvation at different diameter ranges. Relatively high concentrations of SL (5 and 10 µM SL) decrease root length, especially under full nutritional conditions (Fig. 4, and Supplementary Fig. 4).

### Effect of Strigolactone on the soil nutrient content

**Fig. 5:**
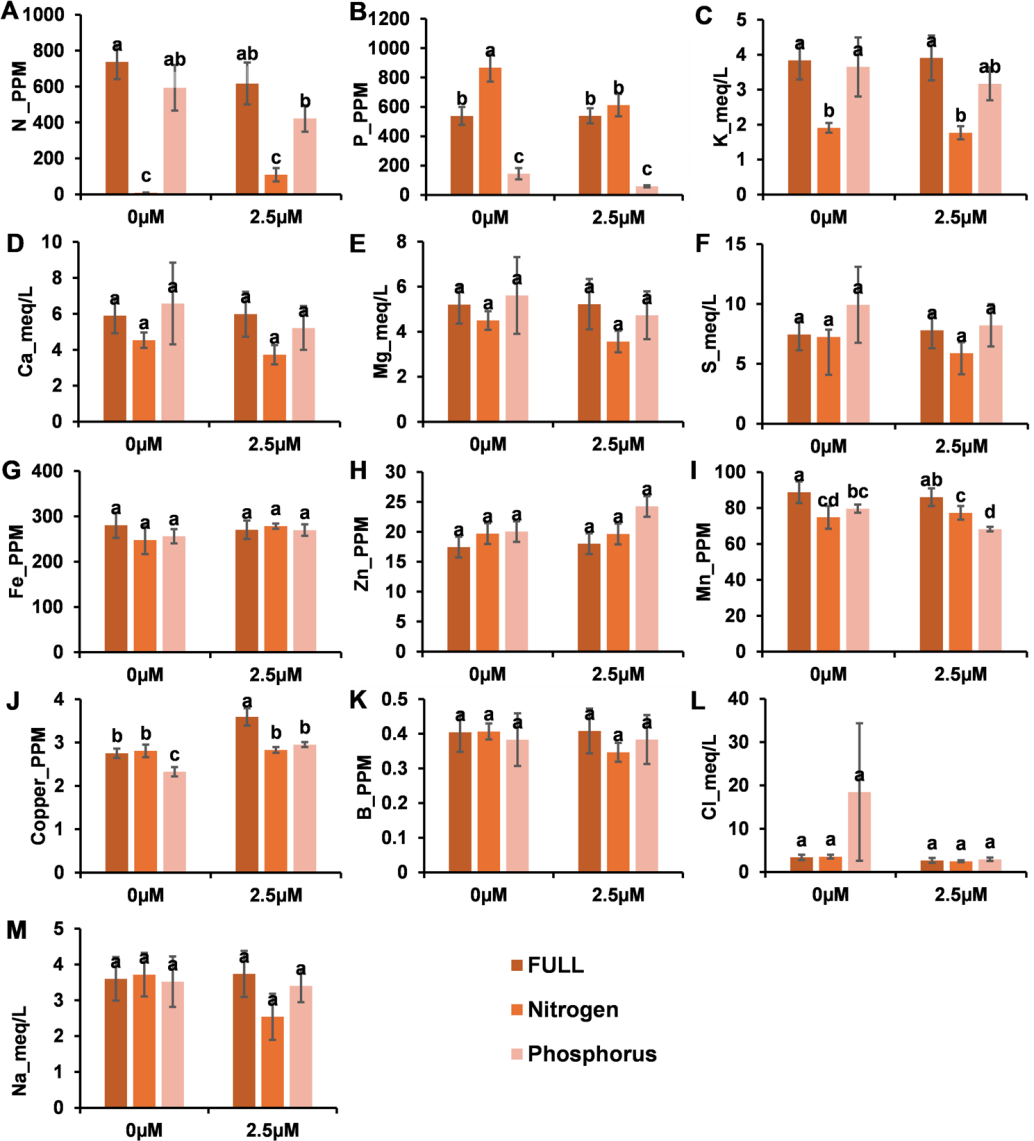
Soil nutrient content in response to SL application (0 and 2.5 µM) under full nutrition, nitrogen deficiency, and phosphorus deficiency. The parameters include (**A**) total N, (**B**) total P, (**C**) K, (**D**) Ca, (**E**) Mg, (**F**) S, (**G**) Fe, (**H**) Zn, (**I**) B, (**J**) Cu, (**K**) Mn, (**L**) Na, and (**M**) Cl. The bars represent the means ± SEs. Different letters denote significant differences among treatments (*p* < 0.05)

As expected, N and P starvation significantly reduced the N and P, respectively, in the soil at 0 µM. Interestingly, SL at 2.5 µM alleviated this reduction by increasing the N in the soil; however, this effect was not statistically significant. SL treatment showed a tendency toward higher nitrogen content in the soil, although this difference was not statistically significant. This indicated the role of soil microbiome attracted by SL, while there was not a source of nitrogen. In contrast, SL decreased the content of P in the soil under P starvation treatment; however, it was also non-significant. **(**Fig. 5 and Supplementary Fig. 5)

The effects of SLs on the contents of some soil nutrients varied with nutrient regime. Under both full nutrition and phosphorus (P) starvation, SLs significantly increased copper (Cu) accumulation. In contrast, phosphorus-starved plants presented a significant decrease in manganese (Mn) content. Under nitrogen (N) starvation, SL application significantly reduced phosphorus levels, suggesting a potential interaction between SL signaling and nutrient uptake or redistribution under stress. There were significant differences between the treatments in terms of Ca, Mg, S, Fe, Zn, B, Na, and Cl. **(**Fig. 5 and Supplementary Fig. 5)

### Effect of Strigolactone on the rhizosphere Microbiome

**Fig. 6:**
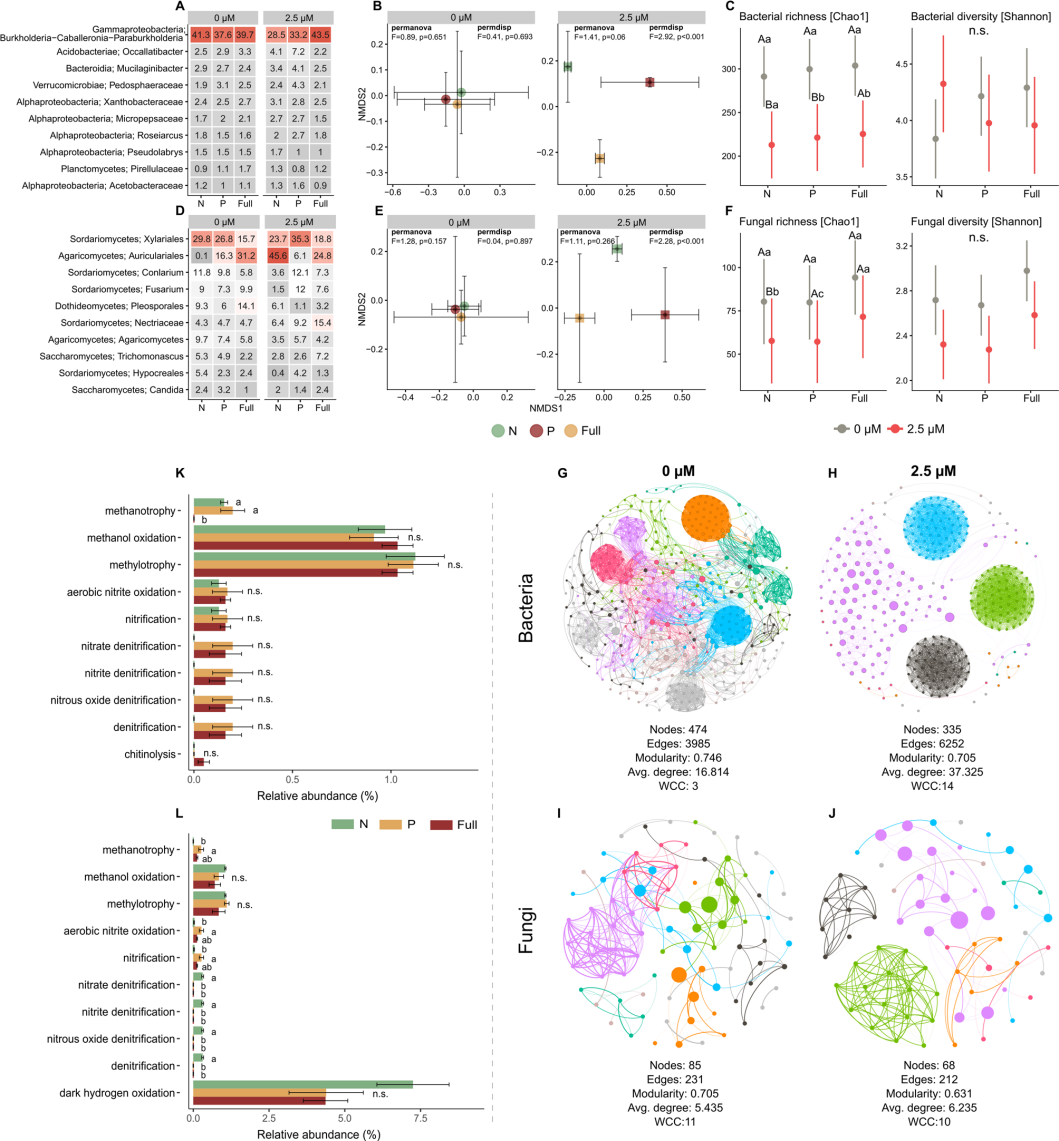
Effects of strigolactone (SL) application with nitrogen (N) or phosphorus (P) deficient conditions or full fertilization on the structure and diversity of the citrus rhizosphere microbiome. Relative abundances of dominant bacterial (**A**) and fungal (**D**) taxa across different nutrient treatments and SL applications. Fertilizer conditions drover higher dissimilarity in bacterial (**B**) and fungal (**E**) community structures in the presence of SL application based on NMDS and PERMANOVA. ASV richness (Chao1) and Shannon diversity of bacterial (**C**) and fungal (**F**) communities under different conditions. Predicted bacterial community functions annotated by FAPROTAX in under 0 µM SL (**K**) or 2.5 µM SL (**L**). Cooccurrence network topologies of bacteria under 0 µM SL (**G**) and 2.5 µM SL (**H**) and fungi under 0 µM SL (**I**) and 2.5 µM SL (**J**). Colors represent modules and node size is based on the betweenness centrality

Overall, *Burkholderia-Caballeronia-Paraburkholderia* represented the most abundant prokaryotic group in the citrus rhizosphere microbiome, showing minimal differences across treatments without strigolactone (SL) application. However, this group was relatively more abundant in the full nutrition + SL treatment than in the nitrogen (N) and phosphorus (P) deficiency treatments **(**Fig. 6**– **A). Despite the limited variation in prokaryotic composition and relative abundance, SL application significantly altered the community structure, particularly affecting the homogeneity of multivariate dispersions (F = 2.92, *p* < 0.001), increasing the separation among the N, P-deficient, and fully nutritive treatments (Fig. 6 – B). This impact was also evident in ASV richness, which significantly decreased across all the treatments under SL influence **(**Fig. 6**– **C), although no significant changes were observed in Shannon diversity.

In contrast, eukaryotic composition exhibited greater variability across treatments, with distinct impacts observed in the presence or absence of SL **(**Fig. 6**– **D). The order *Xylariales* was most abundant, especially under P deficiency with SL application, while *Auriculariales* accounted for a substantial 45.6% of the community under SL influence, although it was nearly absent without SL. Similar to bacterial sequences, higher SL dosages affected the eukaryotic community structure, increasing the degree of distinction across treatments **(**Fig. 6**– **E; permdisp: F = 2.28, *p* < 0.001). This distinction was further captured via alpha diversity analysis, which revealed greater differentiation in eukaryotic richness across treatments post-SL application. Although the ASV richness decreased compared with that in the treatments without SL, only N deficiency significantly decreased **(**Fig. 6**– **F). No significant effects on Shannon diversity were detected in the treatments following SL application.

Functional annotation analysis via FAPROTAX revealed differences among fertilizer treatments for methanotrophy only in the absence of SL **(**Fig. 6**– **K). With SL application, denitrification-related functions were more prominent in the N deficiency treatment, whereas methanotrophy was more pronounced in the P deficiency treatment **(**Fig. 6**–** L).

Finally, the results from the co-occurrence network analysis aligned with the homogeneity of multivariate dispersion analysis and alpha diversity depletion, indicating significant differentiation in the prokaryotic community structure. A reduction in node count and modularity, alongside increases in the average degree and the number of weakly connected components (WCCs), indicated a more defined network structure in rhizosphere communities post-SL application, with a concomitant simplification of node interactions **(**Fig. 6. **– **G-H). In contrast, ITS-based co-occurrence networks showed no substantial shifts in eukaryotic community interaction patterns under SL treatment (Fig. 6-I-J).

## Discussion

Nitrogen (N) and phosphorus (P) are essential macronutrients for plant growth and productivity. However, their availability in soil is often limited. In many regions, such as European countries and California (USA), nitrogen fertilizer use is strictly regulated due to concerns about water contamination [1, 5, 8, 55–57]. On the other hand, phosphorus availability to plants is constrained by its low mobility and ability to be fixed in soil, making P starvation a major challenge in agricultural systems [2, 58].

Conventional fertilization strategies using synthetic N and P fertilizers are increasingly criticized because of their environmental impact. Consequently, interest in the use of organic alternatives or 2.5.

agents to increase nutrient uptake and mitigate deficiency symptoms is increasing [12, 41, 59]. One promising group of such agents is strigolactones (SLs), which are plant hormones that have recently emerged as regulators of root development and nutrient acquisition [35, 36]. In this study, we used rac-GR24, which is widely utilized as a synthetic strigolactone analog in plant studies. While it does not fully replicate the activity of endogenous SLs, it is broadly accepted as a functional tool to investigate SL-related responses.

Despite growing evidence of the roles of SLs in various crops, their impact on citrus remains underexplored. This study investigated the effects of exogenously applied SLs on citrus root system architecture (RSA), biomass allocation, nutrient uptake, and rhizosphere microbiome composition under full nutrition, N starvation, and P starvation.

Root traits such as length, surface area, volume, and number of active tips reflect a plant’s strategy for optimizing nutrient foraging [35, 60–62]. These traits are tightly regulated by hormonal and environmental signals, resulting in a root architecture adapted to available resources [63–65].

Our findings show that the effects of SLs on root development are both concentration- and nutrient status dependent, which is in line with the findings of previous studies [23, 42, 66–68]. Under full nutrient supply, the SL treatment increased the total number of root tips, root hairs, fine tips, and mild-sized lateral root classes while reducing the total root biomass and surface area. SL application increased root tip number while reducing total root biomass under full nutrition. This indicates that SLs promote a more energy-efficient RSA, enhancing exploratory capacity without excessive carbon allocation, consistent with their proposed role as fine-tuning regulators of root plasticity.

Under phosphorus deficiency, SLs elicited the most pronounced root architectural changes. There was a significant increase in the number of root tips and the surface area and volume of fine- and medium-sized roots, enhancing the capacity of the roots to forage P. The concurrent increase in copper (Cu) uptake suggests SL-mediated activation of general nutrient acquisition mechanisms. However, a decrease in manganese (Mn) uptake under SL treatment raises the possibility of nutrient competition or changes in transporter activity, warranting further mechanistic studies. These findings are in agreement with studies reporting enhanced fine root formation and root hair elongation by SLs under P deficiency, likely mediated through interactions with auxin and other signaling molecules [34, 69].

In contrast, SL responses under nitrogen starvation differed markedly. While the surface area and volume of coarse roots increased, possibly to enable exploration of deeper soil layers, the number of fine root tips decreased. This suggests a resource-conserving strategy in which SLs suppress lateral root branching under N stress to prioritize root elongation and reduce metabolic cost. Moreover, SL treatment under N starvation reduced phosphorus accumulation, possibly due to altered root function or disrupted nutrient synergism, reflecting the complex crosstalk between SLs and N signaling pathways [70, 71].

Interestingly, the growth-suppressive effect of SLs on root biomass was observed under full nutrition but not under N or P deficiency. These findings suggest that SLs function differently under optimal versus deficient nutrient conditions, acting as growth regulators in nutrient-rich environments and as facilitators of adaptation under stress [70]. Moreover, the effects of SL were concentration dependent. While 2.5 µM GR24 had minimal effects on overall RSA, higher concentrations (5–10 µM) inhibited root growth, particularly under full nutrition, which is consistent with hormonal feedback inhibition mechanisms [44].

In addition to their effects on root development, SLs significantly influenced nutrient uptake and the rhizosphere microbial community structure. SL application altered soil nutrient profiles by increasing Cu levels under full and P-deficient conditions and reducing Mn and P uptake under specific treatments. These shifts may be driven by GR24-mediated changes in root exudates and microbial activity [62, 72–74].

Although some responses to SL applications, such as increases in nitrogen content and modifications in root characteristics, did not reach statistical significance, we report these as trends because they align with observations from previous studies. For instance, similar tendencies in nutrient accumulation and root system changes under SL or GR24 treatment have been described in *Arabidopsis* and crop species, even when statistical support was limited [75, 76]. Such tendencies may reflect underlying regulatory roles of SLs that could become more evident under different experimental conditions or with greater replication. Nevertheless, we interpret these observations with caution and refrain from drawing definitive conclusions from non-significant results. Under nutrient limitation, SLs restructured the rhizosphere microbiome by reducing amplicon sequence variant (ASV) richness and increasing dispersion, indicating the selective recruitment of microbes. The enrichment of fungal taxa such as *Xylariales* and *Auriculariales* under P deficiency suggests potential roles in increasing P availability. Similarly, the increased microbial denitrification functions under N deficiency imply that SLs influence microbial functional profiles in a nutrient-specific manner.

Network analysis of microbial co-occurrence revealed that GR24 simplified microbial interactions, reducing modularity and increasing connectivity, potentially reflecting a streamlined community that was more efficiently engaged in plant support under stress.

Together, the observed shifts in denitrification and methanotrophy functions were consistent with changes in community structure and dispersion across treatments, especially under GR24 application. While FAPROTAX results rely on taxonomic inference and do not directly measure functional gene abundance, they provide initial insights into how SL may influence nutrient-related microbial functions under stress conditions.

These findings suggest that the dual effects of SL on root development and the rhizosphere microbiome are mechanistically interconnected through root–microbe interactions and nutrient dynamics. GR24-induced modifications in root architecture, such as the stimulation of fine roots and root hairs under phosphorus deficiency or the suppression of root tips under nitrogen deficiency, directly influence rhizodeposition patterns and nutrient fluxes in the soil. At optimal concentrations (e.g., 2.5 µM), GR24 promoted fine root and root hair development, which in turn, can increase the root surface area, the profiles and abundances of exudates released [77], the radial rhizosphere extension, i.e., the habitat for plant- beneficial rhizobacteria involved in nutrient acquisition [78]and microbial activities [79] This expansion of absorptive root zones likely altered nutrient availability and created favorable microhabitats that attracted specific microbial groups, such as Burkholderia-Caballeronia-Paraburkholderia. In contrast, under nitrogen starvation, GR24 suppressed root tip formation and reduced root biomass, which may have limited exudation and altered exudate profiles, leading to decreased ASV richness and the restructuring of microbial networks. Furthermore, GR24-driven changes in soil nutrient pools, including the increase of Cu under P starvation and the significant reduction of Mn, suggest that SL signaling not only modifies nutrient uptake but also indirectly shapes microbial selection. Given the critical role of Mn in enzyme activation and microbial metabolism, its depletion under P deficiency with GR24 may have constrained the abundance of certain microbial taxa while favoring others, contributing to shifts in functional guilds such as denitrifiers and methanotrophs. Collectively, these results indicate that SL likely exerts its effects on microbial attraction and distribution indirectly, by modifying root traits, rhizodeposition patterns, and nutrient availability, thereby restructuring rhizosphere community composition and interactions.

In summary, this study demonstrated that SLs are key modulators of citrus root development and rhizosphere interactions and that their effects are tightly regulated by nutrient status and concentration. SLs enhance root plasticity under P deficiency by promoting fine root proliferation and microbial recruitment, which are conducive to P acquisition. Conversely, under N limitation, SLs suppress branching and may shift root function toward conservation and exploration strategies. These nutrient-specific responses emphasize the complexity of SL signaling and its integration with other nutrient and hormonal pathways. Further studies are needed to elucidate the molecular mechanisms underlying SL–nutrient–microbiome crosstalk and to evaluate the agronomic potential of SL application in enhancing nutrient use efficiency and crop resilience.

## Supplementary Information

Supplementary Material 1.

## Data Availability

The datasets generated and/or analyzed during the current study are available in the NCBI Sequence Read Archive repository, as part of the BioProject Accession PRJNA1290368 (https://www.ncbi.nlm.nih.gov/bioproject/?term=PRJNA1290368).

## Abbreviations

SL: Strigolactone
GR24: Synthetic strigolactone analog
RSA: Root System Architecture
TSA: Total Surface Area
TL: Total Length
TV: Total Volume
TF: Total Forks
TC: Total Crossings
TTs: Total Root Tips
ITS: Internal Transcribed Spacer
ASV: Amplicon Sequence Variant
NMDS: Non-metric Multidimensional Scaling
FAPROTAX: Functional Annotation of Prokaryotic Taxa
WCC: Weakly Connected Component

## References

[1] de Vries W, Posch M, Simpson D, de Leeuw FA, van Grinsven HJ, Schulte-Uebbing LF, Sutton MA, Ros GH. Trends and geographic variation in adverse impacts of nitrogen use in Europe on human health, climate, and ecosystems: A review. Earth-Sci Rev. 2024;253:104789.

[2] Vitousek PM, Porder S, Houlton BZ, Chadwick OA. Terrestrial phosphorus limitation: mechanisms, implications, and nitrogen–phosphorus interactions. Ecol Appl. 2010;20(1):5–15. 10.1890/08-0127.1 20349827

[3] Malhotra H, Vandana; Sharma S, Pandey R. Phosphorus nutrition: plant growth in response to deficiency and excess. In: Hasanuzzaman M, Fujita M, Oku H, Nahar K, Hawrylak-Nowak B, editors. Plant nutrients and abiotic stress tolerance. Singapore: Springer Singapore; 2018. pp. 171–90.

[4] Carranca C, Brunetto G, Tagliavini M. Nitrogen nutrition of fruit trees to reconcile productivity and environmental concerns. Plants. 2018;7(1):4. 10.3390/plants7010004 PMC5874593 29320450

[5] Öquist MG, He H, Bortolazzi A, Nilsson MB, Rodeghiero M, Tognetti R, Ventura M, Egnell G. Nitrogen fertilization increases N2O emission but does not offset the reduced radiative forcing caused by the increased carbon uptake in boreal forests. For Ecol Manag. 2024;556:121739.

[6] Yadav MR, Kumar R, Parihar CM, Yadav RK, Jat SL, Ram H, Meena RK, Singh M, Verma AP, Kumar UJ, Ghosh A. Strategies for improving nitrogen use efficiency: A review. Agric Rev. 2017;38(1):29–40.

[7] Brunelle T, Chakir R, Carpentier A, Dorin B, Goll D, Guilpart N, Maggi F, Makowski D, Nesme T, Roosen J, Tang FH. Reducing chemical inputs in agriculture requires a system change. Commun Earth Environ. 2024;5(1):369.

[8] Yanardağ AB. Damage to Soil and Environment Caused by Excessive Fertilizer Use. Agriculture, Forestry and Aquaculture Sciences. Ankara: Serüven Publishing; 2024. p. 203.

[9] Hussain S, Shafiq I, Skalicky M, Brestic M, Rastogi A, Mumtaz M, Hussain M, Iqbal N, Raza MA, Manzoor S, Liu W. Titanium application increases phosphorus uptake through changes in auxin content and root architecture in soybean (Glycine Max L.). Front Plant Sci. 2021;12:743618. 10.3389/fpls.2021.743618 PMC8631872 34858450

[10] Galindo-Castañeda T, Lynch JP, Six J, Hartmann M. Improving soil resource uptake by plants through capitalizing on synergies between root architecture and anatomy and root-associated microorganisms. Front Plant Sci. 2022;13:827369. 10.3389/fpls.2022.827369 PMC8959776 35356114

[11] Chandel N, Kumar A, Kumar R. Towards sustainable agriculture: integrating agronomic practices, environmental physiology and plant nutrition. International Journal of Plant & Soil Science. 2024;36:492–503.

[12] Sabry N, Soliman.; N G, Rawhya A-H, Ibrahim A, EFFECT OF SOME NA, ORGANIC FERTILIZERS AND HUMIC ACID ON PRODUCTIVITY AND QUALITY OF SUPERIOR GRAPES. (VITIS VINIFERA). J Biol Chem Environ Sci. 2016;11(2):295–317.

[13] Kavamura VN, Robinson RJ, Hughes D, Clark I, Rossmann M, Melo ISd, et al. Wheat dwarfing influences selection of the rhizosphere microbiome. Sci Rep. 2020;10:1452. 10.1038/s41598-020-58402-y. 31996781 10.1038/s41598-020-58402-y PMC6989667

[14] Zwanenburg B, Pospisil T. Structure and activity of strigolactones: new plant hormones with a rich future. Mol Plant. 2013;6:38–62. 10.1093/mp/sss141. 23204499 10.1093/mp/sss141

[15] Koltai H, Dor E, Hershenhorn J, Joel DM, Weininger S, Lekalla S, Shealtiel H, Bhattacharya C, Eliahu E, Resnick N, Barg R. Strigolactones’ effect on root growth and root-hair elongation may be mediated by auxin-efflux carriers. J Plant Growth Regul. 2010;29(2):129–36.

[16] Bouwmeester HJ, Fonne-Pfister R, Screpanti C, De Mesmaeker A. Strigolactones. Plant hormones with promising features. Angew Chem. 2019;58:12778–86. 10.1002/anie.201901626. 31282086 10.1002/anie.201901626

[17] De Cuyper C, Goormachtig S. Strigolactones in the rhizosphere: friend or foe? Mol Plant Microbe Interact. 2017;30:683–90. 10.1094/MPMI-02-17-0051-CR. 28598262 10.1094/MPMI-02-17-0051-CR

[18] Mayzlish-Gati E, De-Cuyper C, Goormachtig S, Beeckman T, Vuylsteke M, Brewer PB, et al. Strigolactones are involved in root response to low phosphate conditions in Arabidopsis. Plant Physiol. 2012;160:1329–41. 10.1104/pp.112.202358. 22968830 10.1104/pp.112.202358 PMC3490576

[19] Foo E, Yoneyama K, Hugill CJ, Quittenden LJ, Reid JB. Strigolactones and the regulation of pea symbioses in response to nitrate and phosphate deficiency. Mol Plant. 2013;6:76–87. 10.1093/mp/sss115. 23066094 10.1093/mp/sss115

[20] Faizan M, Faraz A, Sami F, Siddiqui H, Yusuf M, Gruszka D, et al. Role of strigolactones: signalling and crosstalk with other phytohormones. Open Life Sci. 2020;15:217–28. 10.1515/biol-2020-0022. 33987478 10.1515/biol-2020-0022 PMC8114782

[21] Sun H, Tao J, Gu P, Xu G, Zhang Y. The role of strigolactones in root development. Plant Signal Behav. 2016;11:e1110662. 10.1080/15592324.2015.1110662. 26515106 10.1080/15592324.2015.1110662 PMC4871655

[22] Pandey A, Sharma M, Pandey GK. Emerging roles of Strigolactones in plant responses to stress and development. Front Plant Sci. 2016;7:434. 10.3389/fpls.2016.00434. 27092155 10.3389/fpls.2016.00434 PMC4821062

[23] Smith SM. Witchcraft and destruction. Nature. 2013;504:384–5. 10.1038/nature12843. 24336204 10.1038/nature12843

[24] Zhan Y, Qu Y, Zhu L, Shen C, Feng X, Yu C. Transcriptome analysis of tomato (*Solanum lycopersicum* L.) shoots reveals a crosstalk between auxin and strigolactone. PLoS ONE. 2018;13:e0201124. 10.1371/journal.pone.0201124. 30044859 10.1371/journal.pone.0201124 PMC6059464

[25] Lopez-Raez JA, Charnikhova T, Gomez-Roldan V, Matusova R, Kohlen W, De Vos R, et al. Tomato strigolactones are derived from carotenoids and their biosynthesis is promoted by phosphate starvation. New Phytol. 2008;178:863–74. 10.1111/j.1469-8137.2008.02406.x. 18346111 10.1111/j.1469-8137.2008.02406.x

[26] Koltai H, Dor E, Hershenhorn J, Joel DM, Weininger S, Lekalla S, Shealtiel H, Bhattacharya C, Eliahu E, Resnick N, et al. Strigolactones’ effect on root growth and root-Hair elongation May be mediated by Auxin-Efflux carriers. J Plant Growth Regul. 2009;29:129–36. 10.1007/s00344-009-9122-7.

[27] Koltai H, Prandi C. Strigolactones: past, present and future. Planta. 2016. 10.1007/s00425-016-2541-3. 27141845 10.1007/s00425-016-2541-3

[28] Ćavar S, Zwanenburg B, Tarkowski P. Strigolactones: occurrence, structure, and biological activity in the rhizosphere. Phytochem Rev. 2014;14:691–711. 10.1007/s11101-014-9370-4.

[29] Al-Babili S, Bouwmeester HJ. Strigolactones, a novel carotenoid-derived plant hormone. Annu Rev Plant Biol. 2015;66:161–86. 10.1146/annurev-arplant-043014-114759. 25621512 10.1146/annurev-arplant-043014-114759

[30] Omoarelojie LO, Kulkarni MG, Finnie JF, Van Staden J. Strigolactones and their crosstalk with other phytohormones. Ann Bot. 2019;124:749–67. 10.1093/aob/mcz100. 31190074 10.1093/aob/mcz100 PMC6868373

[31] Santoro V, Schiavon M, Visentin I, Constan-Aguilar C, Cardinale F, Celi L. Strigolactones affect phosphorus acquisition strategies in tomato plants. Plant Cell Environ. 2021;44:3628–42. 10.1111/pce.14169. 34414578 10.1111/pce.14169 PMC9290678

[32] Xie X, Yoneyama K, Yoneyama K. The strigolactone story. Annu Rev Phytopathol. 2010;48:93–117. 10.1146/annurev-phyto-073009-114453. 20687831 10.1146/annurev-phyto-073009-114453

[33] Wang Y, Duran HGS, van Haarst JC, Schijlen EGWM, Ruyter-Spira C, Medema MH, et al. The role of Strigolactones in P deficiency induced transcriptional changes in tomato roots. BMC Plant Biol. 2021;21:349. 10.1186/s12870-021-03124-0. 34301182 10.1186/s12870-021-03124-0 PMC8299696

[34] Marek M, Aleksandra M, Damian G. The role of Strigolactones in Nutrient-Stress responses in plants. Int J Mol Sci. 2013;14:9286–304. 10.3390/ijms14059286. 23629665 10.3390/ijms14059286 PMC3676783

[35] Soliman S, Wang Y, Han Z, Pervaiz T, El-Kereamy A. Strigolactones in plants and their interaction with the ecological microbiome in response to abiotic stress. Plants. 2022. 10.3390/plants11243499. 36559612 10.3390/plants11243499 PMC9781102

[36] Soliman S, Wang Y, Han Z, El-kereamy A. The attraction of Apple rhizosphere microorganisms and changing leaf characteristics by Strigolactone. Horticulturae. 2023;9:528.

[37] Jangde S, Shruti S, Dwivedi P. Interpreting the Genetic Symphony: Strigolactones and Their Regulatory Effect on Plant Growth and Development. In Plant Growth Regulators: Resilience for Sustainable Agriculture. Singapore: Springer Nature Singapore; 2024. p. 95–113.

[38] Javed S, Chai X, Wang X, Xu S. The phytohormones underlying the plant lateral root development in fluctuated soil environments. Plant Soil. 2024;505(1):101–14.

[39] Lucido A, Andrade F, Basallo O, Eleiwa A, Marin-Sanguino A, Vilaprinyo E, Sorribas A, Alves R. Modeling the effects of strigolactone levels on maize root system architecture. Front Plant Sci. 2024;14:1329556. 10.3389/fpls.2023.1329556 PMC10808495 38273953

[40] Saleem M, Law AD, Sahib MR, Pervaiz ZH, Zhang Q. Impact of root system architecture on rhizosphere and root microbiome. Rhizosphere. 2018;6:47–51. 10.1016/j.rhisph.2018.02.003.

[41] Soliman SN, Abdel-Hamid N, Aly R, Ibrahim MF, Nasser MA. Application of Exogenous Polyols, Amino Acids and Girdling to Improve (Vitis vinifera L.) Crimson Seedless cv. Berries Coloration, and Postharvest Quality. Am Eurasian J Agric Environ Sci. 2023;23(2):70–80.

[42] Marro N, Lidoy J, Chico MA, Rial C, Garcia J, Varela RM, Macias FA, Pozo MJ, Janouskova M, Lopez-Raez JA, Strigolactones. New players in the nitrogen-phosphorus signalling interplay. Plant Cell Environ. 2022;45:512–27. 10.1111/pce.14212. 34719040 10.1111/pce.14212

[43] Andreo-Jimenez B, Ruyter-Spira C, Bouwmeester HJ, Lopez-Raez JA. Ecological relevance of Strigolactones in nutrient uptake and other abiotic stresses, and in plant-microbe interactions below-ground. Plant Soil. 2015;394:1–19. 10.1007/s11104-015-2544-z.

[44] Min Z, Li R, Chen L, Zhang Y, Li Z, Liu M, et al. Alleviation of drought stress in grapevine by foliar-applied strigolactones. Plant Physiol Biochem. 2019;135:99–110. 10.1016/j.plaphy.2018.11.037. 30529172 10.1016/j.plaphy.2018.11.037

[45] Hoagland DR, Arnon DI. The water-culture method for growing plants without soil. 2nd ed. Berkeley: University of California, College of Agriculture, Agricultural Experiment Station. 1950. (Circular No. 347).

[46] Hoagland DR, Arnon DI. The water-culture method for growing plants without soil. 347 ed. Berkeley, Calif.: University of California, College of Agriculture, Agricultural Experiment Station.:; 1938.

[47] Wang WN, Min Z, Wu JR, Liu BC, Xu XL, Fang YL, et al. Physiological and transcriptomic analysis of cabernet sauvginon (*Vitis vinifera* L.) reveals the alleviating effect of exogenous strigolactones on the response of grapevine to drought stress. Plant Physiol Biochem. 2021;167:400–9. 10.1016/j.plaphy.2021.08.010. 34411779 10.1016/j.plaphy.2021.08.010

[48] Edwards J, Johnson C, Santos-Medellín C, Lurie E, Podishetty NK, Bhatnagar S, Eisen JA, Sundaresan V. Structure, variation, and assembly of the root-associated microbiomes of rice. Proc Natl Acad Sci. 2015;112(8):E911–20. 10.1073/pnas.1414592112 PMC4345613 25605935

[49] Illumina I. 16S Metagenomic sequencing library preparation. Preparing 16S ribosomal RNA gene amplicons for the illumina MiSeq system. 2013;1:28.

[50] Apprill A, McNally S, Parsons R, Weber L. Minor revision to V4 region SSU rRNA 806R gene primer greatly increases detection of SAR11 bacterioplankton. Aquat Microb Ecol. 2015;75:129–37.

[51] McMurdie PJ, Holmes S. phyloseq: an R package for reproducible interactive analysis and graphics of microbiome census data. PloS One. 2013;8(4):e61217. 10.1371/journal.pone.0061217 PMC3632530 23630581

[52] Andersen KS, Kirkegaard RH, Karst SM, Albertsen M. ampvis2: an R package to analyse and visualise 16S rRNA amplicon data. BioRxiv; 2018. p. 299537.

[53] Liu C, Cui Y, Li X, Yao M. microeco: an R package for data mining in microbial community ecology. FEMS Microbiol Ecol. 2021;97(2):fiaa255. 10.1093/femsec/fiaa255 33332530

[54] Louca S, Parfrey LW, Doebeli MJS. Decoupling function and taxonomy in the global ocean microbiome. Science. 2016;353:1272–7. 27634532 10.1126/science.aaf4507

[55] Cahn M, Smith R, Melton F. Field evaluations of the CropManage decision support tool for improving irrigation and nutrient use of cool season vegetables in California. Agric Water Manag. 2023;287:108401.

[56] Valenzuela HJN. Optimizing the nitrogen use efficiency in vegetable crops. Nitrogen. 2024;5:106–43.

[57] Xing Y, Wang X. Impact of agricultural activities on climate change: A review of greenhouse gas emission patterns in field crop systems. Plants. 2024;13(16):2285. 10.3390/plants13162285 PMC11360188 39204720

[58] Oliverio AM, Bissett A, McGuire K, Saltonstall K, Turner BL, Fierer N. The role of phosphorus limitation in shaping soil bacterial communities and their metabolic capabilities. MBio. 2020;11(5):10–128. 10.1128/mBio.01718-20 PMC7593963 33109755

[59] Lu W, Hao Z, Ma X, Gao J, Fan X, Guo J, et al. Effects of different proportions of organic fertilizer replacing chemical fertilizer on soil nutrients and fertilizer utilization in Gray desert soil. Agronomy. 2024;14:228.

[60] Lopez-Raez JA, Shirasu K, Foo E. Strigolactones in plant interactions with beneficial and detrimental organisms: the Yin and Yang. Trends Plant Sci. 2017;22:527–37. 10.1016/j.tplants.2017.03.011. 28400173 10.1016/j.tplants.2017.03.011

[61] Jansson JK, Hofmockel KS. Soil microbiomes and climate change. Nat Rev Microbiol. 2020;18:35–46. 10.1038/s41579-019-0265-7. 31586158 10.1038/s41579-019-0265-7

[62] Dubey A, Malla MA, Khan F, Chowdhary K, Yadav S, Kumar A, et al. Soil microbiome: a key player for conservation of soil health under changing climate. Biodivers Conserv. 2019;28(8–9):2405–29. 10.1007/s10531-019-01760-5.

[63] Bosemark NO. The influence of nitrogen on root development. Physiol Plant. 1954;7(3).

[64] Sun X, Chen F, Yuan L, Mi GJP. The physiological mechanism underlying root elongation in response to nitrogen deficiency in crop plants. Planta. 2020;251:84. 32189077 10.1007/s00425-020-03376-4

[65] Tang Q, Ma Y, Zhao L, Song Z, Yin Y, Wang G, et al. Effects of water and nitrogen management on root morphology, nitrogen metabolism enzymes, and yield of rice under drip irrigation. Agronomy. 2023;13:1118.

[66] Danish S, Hareem M, Dawar K, Naz T, Iqbal MM, Ansari MJ, Salmen SH, Datta R. The role of strigolactone in alleviating salinity stress in chili pepper. BMC Plant Biol. 2024;24(1):209. 10.1186/s12870-024-04900-4 PMC10960418 38519997

[67] Gong J, Wang R, Liu B, Zhu T, Li H, Long S, et al. Regulatory mechanism of Strigolactone in tall fescue to low-light stress. Plant Physiol Biochem. 2024;215:109054. 10.1016/j.plaphy.2024.109054. 39163653 10.1016/j.plaphy.2024.109054

[68] Kapoor RT, Alam P, Chen Y, Ahmad P. Strigolactones in plants: from development to abiotic stress management. J Plant Growth Regul. 2024;43:903–19. 10.1007/s00344-023-11148-z.

[69] Sun H, Bi Y, Tao J, Huang S, Hou M, Xue R, Liang Z, Gu P, Yoneyama K, Xie X, Shen Q. Strigolactones are required for nitric oxide to induce root elongation in response to nitrogen and phosphate deficiencies in rice. Plant Cell Environ. 2016;39(7):1473–84. 10.1111/pce.12709 27194103

[70] Marzec M, Melzer M. Regulation of root development and architecture by strigolactones under optimal and nutrient deficiency conditions. Int J Mol Sci. 2018;19:1887. 29954078 10.3390/ijms19071887 PMC6073886

[71] Stassen MJJ, Hsu SH, Pieterse CMJ, Stringlis IA. Coumarin communication along the microbiome-root-shoot axis. Trends Plant Sci. 2021;26:169–83. 10.1016/j.tplants.2020.09.008. 33023832 10.1016/j.tplants.2020.09.008

[72] Berendsen RL, Pieterse CM, Bakker PA. The rhizosphere microbiome and plant health. Trends Plant Sci. 2012;17:478–86. 10.1016/j.tplants.2012.04.001. 22564542 10.1016/j.tplants.2012.04.001

[73] Chaudhari D, Rangappa K, Das A, Layek J, Basavaraj S, Kandpal BK, et al. Pea (*Pisum sativum* L.) plant shapes its rhizosphere microbiome for nutrient uptake and stress amelioration in acidic soils of the North-East region of India. Front Microbiol. 2020;11:968. 10.3389/fmicb.2020.00968. 32582047 10.3389/fmicb.2020.00968 PMC7283456

[74] Trivedi P, Leach JE, Tringe SG, Sa T, Singh BK. Plant-microbiome interactions: from community assembly to plant health. Nat Rev Microbiol. 2020;18:607–21. 10.1038/s41579-020-0412-1. 32788714 10.1038/s41579-020-0412-1

[75] Ruyter-Spira C, Kohlen W, Charnikhova T, van Zeijl A, van Bezouwen L, de Ruijter N, et al. Physiological effects of the synthetic Strigolactone analog GR24 on root system architecture in arabidopsis: another belowground role for Strigolactones? Plant Physiol. 2011;155:721–34. 10.1104/pp.110.166645. 21119044 10.1104/pp.110.166645 PMC3032462

[76] Kohlen W, Charnikhova T, Liu Q, Bours R, Domagalska MA, Beguerie S, et al. Strigolactones are transported through the xylem and play a key role in shoot architectural response to phosphate deficiency in nonarbuscular mycorrhizal host Arabidopsis. Plant Physiol. 2011;155:974–87. 10.1104/pp.110.164640. 21119045 10.1104/pp.110.164640 PMC3032481

[77] Badri D.V., Vivanco J.M.J.P. Regulation and function of root exudates. cell; Environ. 2009;32:666–81. 10.1111/j.1365-3040.2008.01926.x 19143988

[78] Holz M, Zarebanadkouki M, Kuzyakov Y, Pausch J, Carminati A. Root hairs increase rhizosphere extension and carbon input to soil. Ann Bot. 2018;121(1):61–9. 10.1093/aob/mcx127 PMC5786240 29267846

[79] Zhang X, Kuzyakov Y, Zang H, Dippold MA, Shi L, Spielvogel S, et al. Rhizosphere hotspots: root hairs and warming control microbial efficiency, carbon utilization and energy production. Biochemistry. 2020;148:107872.

