# Supplementary material for: Strigolactone GR24 modulates citrus root architecture and rhizosphere microbiome under nitrogen and phosphorus deficiency
Source: BMC Plant Biol. 2025 Nov 14;25:1569. doi: 10.1186/s12870-025-07515-5 (PMC12619333; doi:10.1186/s12870-025-07515-5)
Supplement: Supplementary file 1 — Supplementary Material 1. [file 12870_2025_7515_MOESM1_ESM.pdf]

Supplementary figures of root analysis

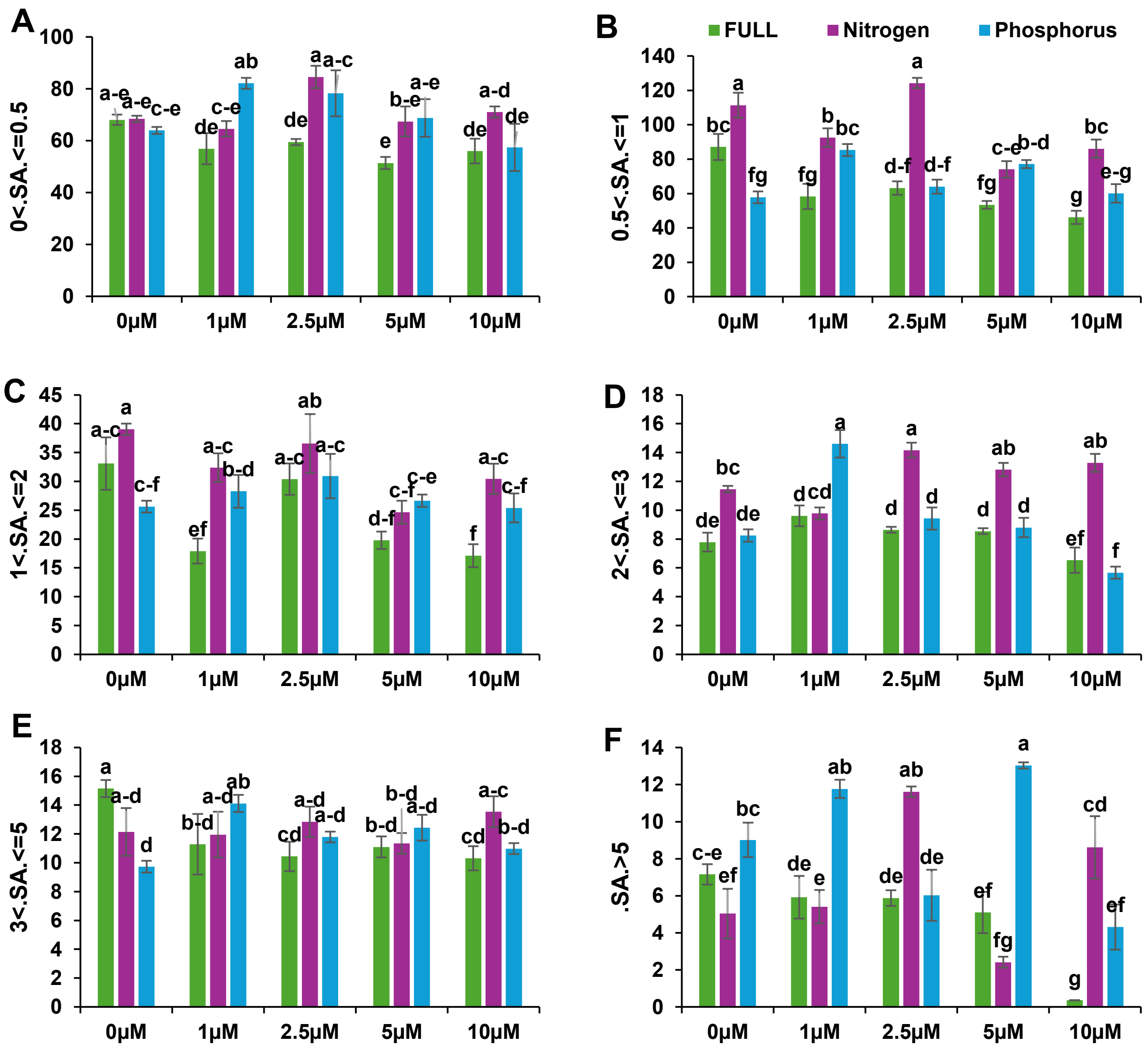

Figure 1. Root surface area (SA) across diameter classes (A–F: 0–0.5 mm to >5 mm) under SL application (0–10 μM) and nutrient treatments. Bars represent means ± SE. Different letters denote significant differences among treatments ($p < 0.05$).

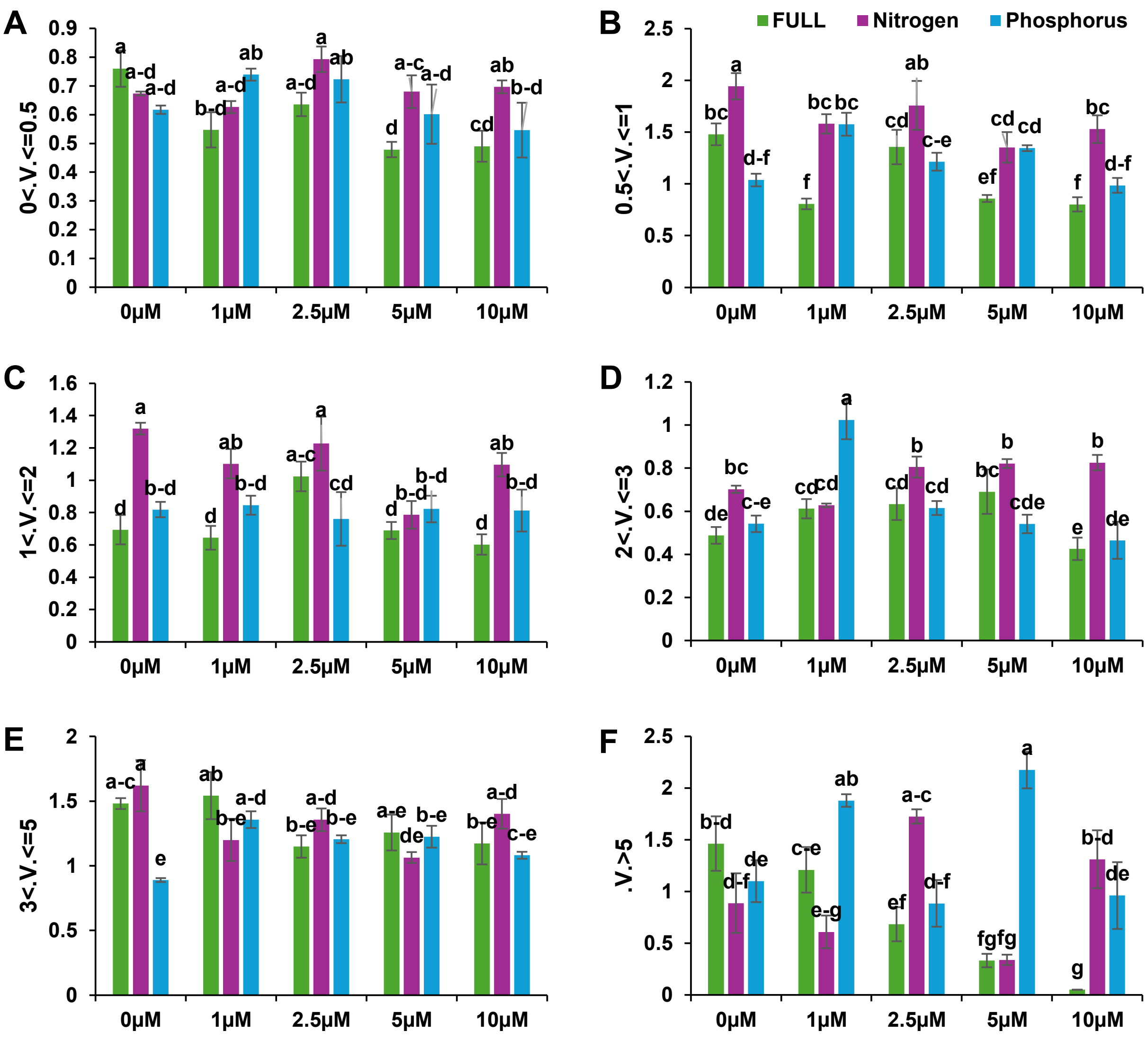

Figure 2. Root volume (V) across root diameter classes (A–F: 0–0.5 mm to >5 mm) under SL treatments at 0, 1, 2.5, 5, and 10 μM, across different nutrient conditions. Bars represent means ± SE. Letters indicate statistically significant differences ($p < 0.05$).

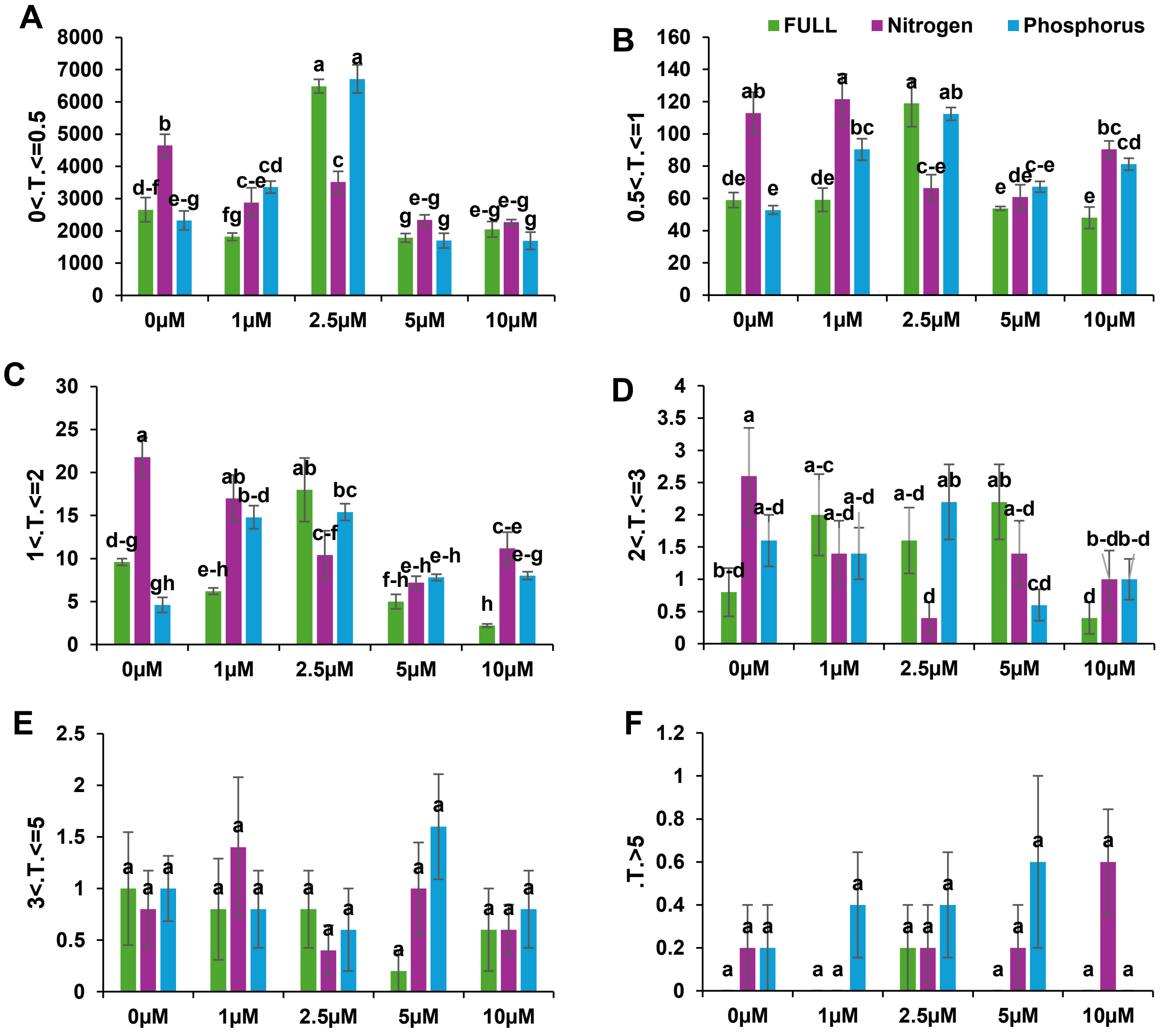

Figure 3. Number of root tips per diameter class (A–F: 0–0.5 mm to >5 mm) under increasing SL concentrations (0 to 10 µM) and nutrient stress. Bars represent means ± SE. Different letters denote significant differences ($p < 0.05$).

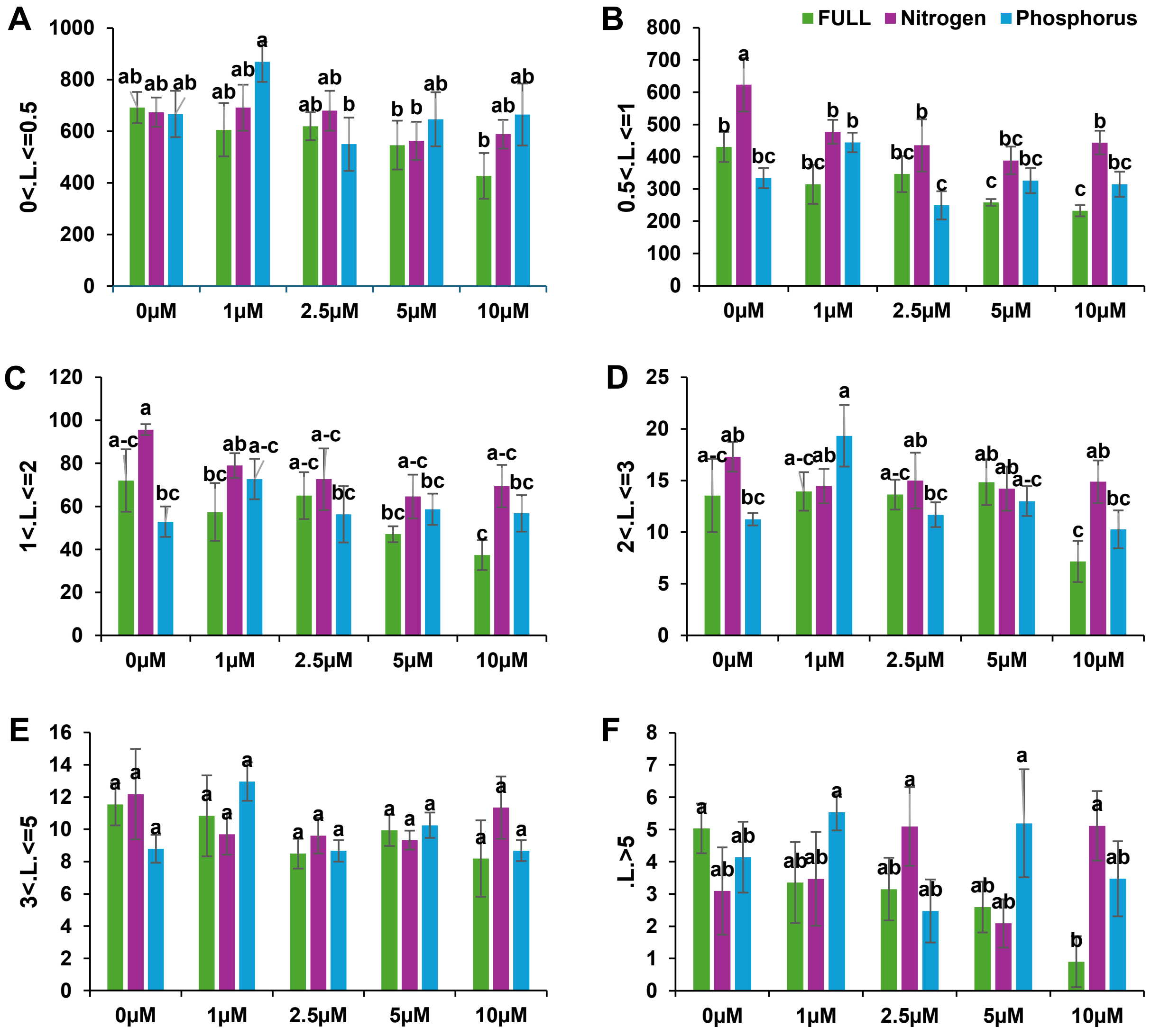

Figure 4. Root length distribution across diameter classes (A–F: 0–0.5 mm to >5 mm) in response to SL concentrations of 0, 1, 2.5, 5, and 10 μM under full nutrition, N and P deficiency. Bars show means ± SE. Different letters indicate significant differences ($p < 0.05$).

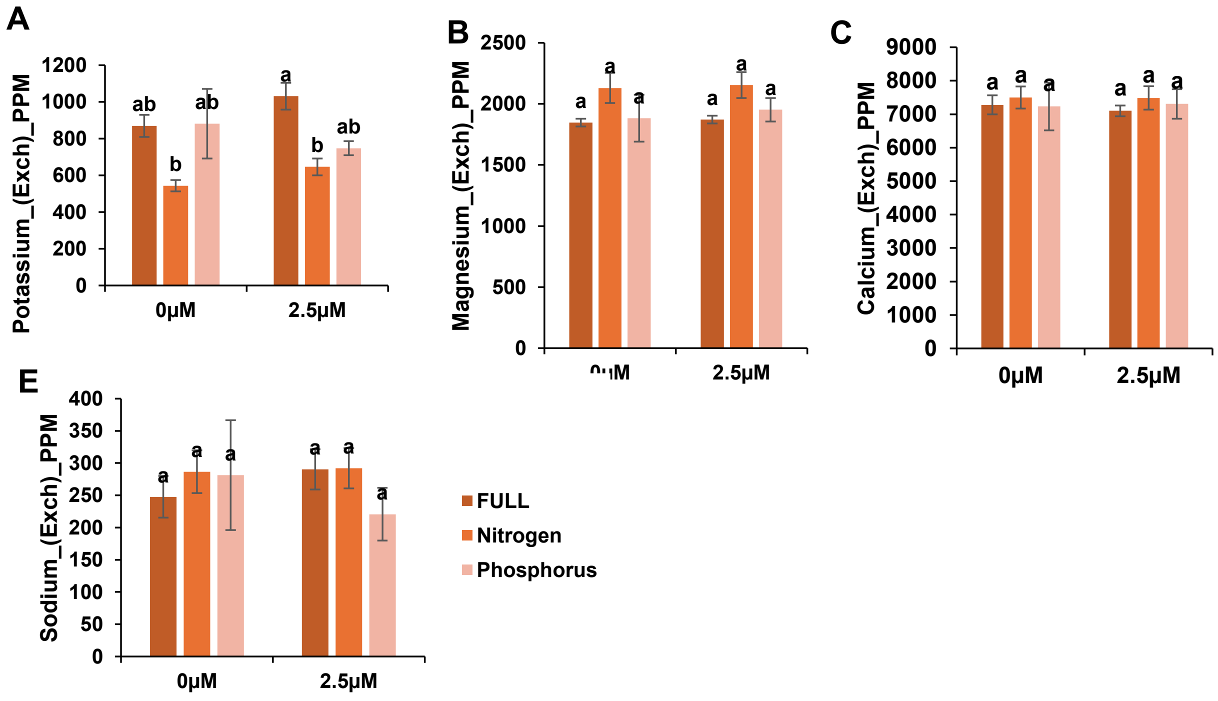

Figure 5. Soil nutrient content in response to strigolactone application (0 to 10 µM) under full nutrition, N deficiency, and P deficiency. This figure complements the main soil nutrient data in Figure 4. Bars represent means ± SE. Different letters indicate significant differences ($p < 0.05$).
